# Social patterning of telephone health-advice for diarrhoea and vomiting: analysis of 24 million telehealth calls in England

**DOI:** 10.1016/j.jinf.2018.09.008

**Published:** 2019-02

**Authors:** Natalie L. Adams, Tanith C. Rose, Alex J. Elliot, Gillian Smith, Roger Morbey, Paul Loveridge, James Lewis, Gareth Studdard, Mara Violato, Sarah J. O'Brien, Margaret Whitehead, David C. Taylor-Robinson, Jeremy I. Hawker, Benjamin Barr

**Affiliations:** a NIHR Health Protection Research Unit in Gastrointestinal Infections, Liverpool, UK; b Department of Public Health and Policy, University of Liverpool, UK; c National Infection Service, Public Health England, London, UK; d Real-time Syndromic Surveillance Team, Field Service, National Infection Service, Public Health England, Birmingham, UK; e NIHR Health Protection Research Unit in Emergency Preparedness and Response, London, UK; f Health Economics Research Centre, University of Oxford, Oxford, UK; g Emergency Response Department, Science and Technology, Health Protection Directorate, Public Health England, Porton Down, Salisbury, UK; h NHS England, West Midlands Integrated Urgent Care, Birmingham, UK; i National Infection Service, Field Service, Public Health England, Birmingham, UK

**Keywords:** Diarrhoea, Vomiting, Inequalities, Syndromic surveillance

## Abstract

**Objectives:**

Gastrointestinal (GI) infections are common and most people do not see a physician. There is conflicting evidence of the impact of socioeconomic status (SES) on risk of GI infections. We assessed the relationship between SES and GI calls to two National Health Service (NHS) telephone advice services in England.

**Methods:**

Over 24 million calls to NHS Direct (2010–13) and NHS 111 (2013–15) were extracted from Public Health England (PHE) syndromic surveillance systems. The relationship between SES and GI calls was assessed using generalised linear models (GLM).

**Results:**

Adjusting for rurality and age-sex interactions, in NHS Direct, children in disadvantaged areas were at lower risk of GI calls; in NHS 111 there was a higher risk of GI calls in disadvantaged areas for all ages (0–4 years RR 1.27, 95% CI 1.25–1.29; 5–9 years RR 1.43, 95% CI 1.36–1.51; 10–14 years RR 1.36, 95% CI 1.26–1.41; 15–19 years RR 1.59, 95% CI 1.52–1.67; 20–59 years RR 1.50, 95% CI 1.47–1.53, 60 years and over RR 1.12, 95% CI 1.09–1.14).

**Conclusions:**

Disadvantaged areas had higher risk of GI calls in NHS 111. This may relate to differences in exposure or vulnerability to GI infections, or propensity to call about GI infections.

## Highlights

• Disadvantaged areas were associated with higher risk of gastrointestinal infection (GI) calls to the National Health Service (NHS) telephone advice services in England.
• This trend was seen across age groups.
• This may reflect differential exposure or vulnerability to GI infections by socioeconomic status.
• It may also reflect differential propensity to call about GI infections by socioeconomic status.

## Introduction

Gastrointestinal (GI) infections are common in the population, leading to diarrhoea and vomiting as well as more serious health problems. Previous estimates suggest that around 25% of people in the UK suffer an episode of infectious intestinal disease (IID) per year and that foodborne illness in England and Wales costs around £1.5 billion annually.^1^ Many infections are known to vary by social group however the role of socioeconomic inequalities in risk of GI infection in high income countries, such as in the UK, is not well understood, with studies presenting conflicting findings.^2^ Most cases of GI infection are not identified by routine surveillance systems as most GI infections are self-limiting; it is estimated that there are 147 cases in the community for every one case that is reported to national surveillance, such as via laboratory reports.^1^ This level of underreporting presents a challenge to understanding the relationship between infection and socioeconomic status (SES) due to the potential bias in healthcare seeking behaviour within certain groups of the population. It is therefore important to attempt to capture potential inequalities in GI infections particularly amongst individuals who would not be captured in formal surveillance systems. Telephone helplines are underutilised for GI surveillance but potentially give a closer reflection of true community incidence than other routine measures.

This study therefore aims to investigate the relationship between SES and calls to the national telephone helplines for health advice with symptoms of diarrhoea and vomiting; defined as GI calls. Our findings will deepen the understanding of socioeconomic and socio-demographic inequalities of GI infections in the UK.

## Methods

### Data, setting and source

An analysis of calls made to the two National Health Service (NHS) telephone helplines for health advice in England, NHS Direct (October 2010 to July 2013) and its successor NHS 111 (October 2013 to July 2015), was undertaken to explore the role of socioeconomic status on reporting of GI symptoms. These systems provide advice delivered to individuals over the telephone as opposed to face-to-face consultation. NHS Direct covered England and Wales and NHS 111 covered England only. Therefore, for comparability, the NHS Direct dataset was restricted to calls from England only. All calls with a valid postcode district in England and reported to the PHE syndromic surveillance systems were included. A postcode district is the first half of the postcode and covers approximately 10,000 households.^3^ Data were aggregated to the number of calls per month, postcode district, age group and gender. Population by age group and gender for each postcode district were linked with the call data to allow for population-level comparisons.

An observational study design was used to assess socioeconomic inequalities in calls classified as ‘diarrhoea’ and/or ‘vomiting’ (the only codes used for acute GI infection), which were collectively defined as GI calls, with calls made about other symptoms, defined as non-GI calls. Symptoms were self-reported by callers using the national telehealth services. Data from NHS Direct and NHS 111 were extracted from the Public Health England (PHE) NHS Direct/111 syndromic surveillance systems, based upon data routinely collected and used by PHE for public health surveillance from October 2010 to July 2015.^4^^,^ ^5^ Due to the changeover between systems, no data were extracted in August or September 2013 to allow for potential drop-off and uptake of reporting across the two systems.

This study falls under the existing PHE permissions under Section 251 of the NHS Act 2006, however no identifiable data were used in this study therefore specific ethical approval was not required. The data used were routine syndromic surveillance data collected by PHE to undertake the real-time syndromic surveillance service; permission to use the syndromic surveillance datasets for this research was obtained from the PHE/NHS 111 steering group.

### Outcome and exposures

The primary outcome of interest for this study was GI calls. The primary exposure of interest was an area-level measure of SES, the Index of Multiple Deprivation 2010 (IMD 2010)^6^ generated using the population-weighted mean IMD score for each postcode district which was assigned to each call, categorised into IMD quintiles. The Office for National Statistics Rural Urban Classification^7^ was used to assign the proportion of the population classified as urban for each postcode district. Other covariates of interest included in the analysis were age (coded as 0–4, 5–9, 10–15, 16–19, 20–59 and 60 years and over); sex (male/female) and urban decile (proportion of population classed as urban, operationalised as deciles).

### Analysis strategy

Analyses were conducted in R (version 3.3.1). A descriptive analysis of the SES of GI calls compared to non-GI calls was undertaken. Crude incidence rates, incidence differences and incidence ratios by SES were calculated, stratified by gender and age group. The main analysis explored the relationship between GI calls and SES using a generalised linear model (GLM) with a Poisson family and log-link function. To model the call rate, the log of the population in each postcode district, age group and gender was included in the model as an offset. Postcode districts may contain households with no resident population. Due to some age groups within postcode districts having a population of zero, these were excluded from the main analysis (n = 1357, 0.1%). Separate analyses were undertaken for NHS Direct and NHS 111 as it was not justified to pool the results due to differences in rates between two systems as a result of NHS 111 also acting as an out of hours general practice (GP) service which increased call rates.^4^ The multivariable model described above was then fitted with SES (IMD quintile) as the exposure variable and calls as the outcome variable, adjusting for the potential confounders (age group, sex and urban decile and interactions between age and sex, and age and IMD quintile).Previous literature suggested that the relationship between SES and GI risk may vary across the life course,^8^ so an interaction term between IMD quintile and age group was included in the model. Risk ratios and 95% confidence intervals (CIs) were estimated.

### Robustness tests

To assess whether the recoding of postcode districts with no population affected the results, postcode districts with a population of zero were recoded to one and included in the analysis. Secondly, to test whether there was a significant trend across levels of deprivation and rurality, the analysis was repeated using IMD Score and the proportion of the population classed as urban as continuous variables. Due to changing protocols in NHS Direct which meant symptom information was unavailable for infants < 1 year of age after November 2011, sensitivity analysis excluding calls regarding infants < 1 was conducted for both NHS Direct and NHS 111. To assess the potential role of healthcare access, the average (mean) distance to a GP^9^ within each postcode district was added to the model.

## Results

A total of 24,214,879 calls were included in the study over the six year period (NHS Direct n = 7,874,257; NHS 111 n = 16,340,622). Of these, 6.0% (n = 1,450,843) were classed as GI calls (NHS Direct: 6.5%, 513,363; NHS 111: 5.7%, n = 937,480). Age was missing for 431,239 records (1.8%) and sex for 314,982 records (1.3%). After excluding records with missing data, 23,762,217 calls remained.

**Fig. 1:**
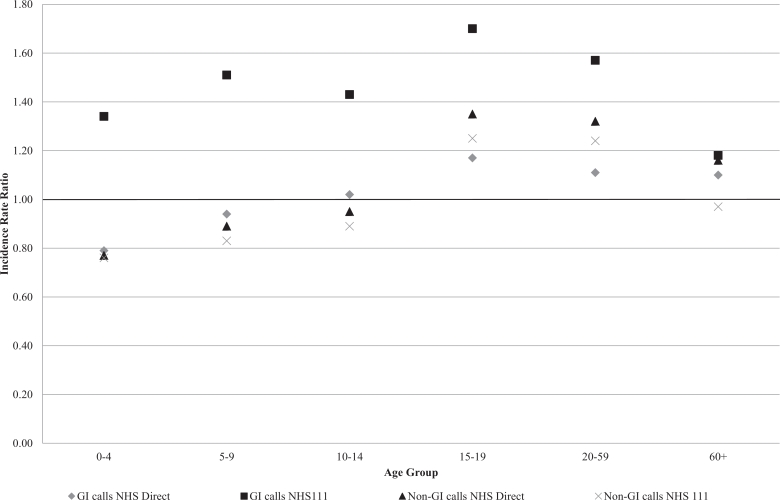
Incidence rate ratios for most disadvantaged compared to least disadvantaged by age group and system. Footnote: GI – Gastrointestinal infection; NHS – National Health Service.

Call rates for all age groups for both GI and non-GI calls were considerably higher in NHS 111 (Fig. 1). In NHS Direct, crude incident rate ratios for GI and non-GI calls show a relationship between deprivation and age, with significantly higher rates for adults (age 15+) in disadvantaged areas and significantly lower rates for children (aged 0–4 for GI calls and 0–14 for non-GI calls) in disadvantaged areas. In NHS 111, crude GI call rates were significantly higher for both children and adults in disadvantaged areas; however, non-GI calls amongst children (aged 0–14) were significantly lower in the most disadvantaged areas compared to the least disadvantaged and significantly higher amongst adults (aged 15–59) in disadvantaged areas.

Data were aggregated to postcode district, age group and gender for the main analysis. The aggregated data used in the regression analysis consisted of 49,970 postcode district, age and gender groups.

As there was a significant interaction between age group and IMD quintile, Table 1 presents the risk ratio for IMD in each age group for NHS Direct and NHS 111 derived from the interaction terms, adjusting for sex and proportion of the population classed as urban. In NHS Direct, there was a statistically significant lower risk of calling with GI symptoms amongst the most disadvantaged compared to the least disadvantaged children under 10 years of age but there was no significant difference in age for adults. In NHS 111, there was a statistically significant higher risk of calling with GI symptoms amongst the most disadvantaged compared to the least disadvantaged. The trend across quintiles was clearer in NHS 111, with the risk statistically significantly higher in the most disadvantaged compared to the least disadvantaged in all age-groups.

**Table 1:** Multivariable regression analysis presenting main effect with interaction terms for GI calls in each age group by system.

		NHS Direct	NHS 111
Age group	IMD Quintile	RR^a^ (95% CI)	RR^a^ (95% CI)
**0–4**	**1** (Least disadvantaged)	1.00 (reference)	1.00 (reference)
	**2**	**0.98 (0.96–1.00)**	**1.20 (1.18–1.22)**
	**3**	**0.93 (0.92–0.95)**	**1.37 (1.35–1.39)**
	**4**	**0.86 (0.84–0.87)**	**1.34 (1.31–1.36)**
	**5** (Most disadvantaged)	**0.72 (0.71–0.74)**	**1.27 (1.25–1.29)**
**5–9**	**1** (Least disadvantaged)	1.00 (reference)	1.00 (reference)
	**2**	0.98 (0.93−1.04)	**1.16 (1.11**−**1.22)**
	**3**	1.01 (0.96−1.07)	**1.38 (1.32**−**1.45)**
	**4**	0.96 (0.91−1.01)	**1.44 (1.37**−**1.51)**
	**5** (Most disadvantaged)	**0.85 (0.80–0.90)**	**1.43 (1.36**−**1.51)**
**10**−**14**	**1** (Least disadvantaged)	1.00 (reference)	1.00 (reference)
	**2**	**1.09 (1.00–1.18)**	**1.19 (1.11**−**1.28)**
	**3**	**1.11 (1.03–1.21)**	**1.36 (1.27**−**1.46)**
	**4**	1.08 (0.99–1.17)	**1.36 (1.27**−**1.46)**
	**5** (Most disadvantaged)	0.92 (0.85−1.01)	**1.36 (1.26**−**1.30)**
**15**−**19**	**1** (Least disadvantaged)	1.00 (reference)	1.00 (reference)
	**2**	**1.07 (1.01**−**1.13)**	**1.24 (1.18**−**1.30)**
	**3**	**1.12 (1.06**−**1.18)**	**1.47 (1.41**−**1.54)**
	**4**	**1.12 (1.06**−**1.18)**	**1.54 (1.47**−**1.61)**
	**5** (Most disadvantaged)	1.05 (0.99−1.11)	**1.59 (1.52**−**1.67)**
**20**−**59**^b^	**1** (Least disadvantaged)	1.00 (reference)	1.00 (reference)
	**2**	**1.02 (1.00**−**1.04)**	**1.23 (1.21**−**1.26)**
	**3**	**1.02 (1.00**−**1.04)**	**1.42 (1.40**−**1.45)**
	**4**	**1.04 (1.02**−**1.06)**	**1.46 (1.44**−**1.49)**
	**5** (Most disadvantaged)	1.01 (0.99−1.03)	**1.50 (1.47**−**1.53)**
**60+**	**1** (Least disadvantaged)	1.00 (reference)	1.00 (reference)
	**2**	1.00 (0.97−1.03)	**1.13 (1.11**−**1.15)**
	**3**	**1.03 (1.00**−**1.06)**	**1.26 (1.23**−**1.28)**
	**4**	**1.03 (1.00**−**1.06)**	**1.19 (1.17**−**1.22)**
	**5** (Most disadvantaged)	0.99 (0.96−1.03)	**1.12 (1.09**−**1.14)**

GI – Gastrointestinal infection; CI – Confidence interval; NHS – National Health Service

a Linear combination of main effect + interaction between age and IMD quintile, adjusted for sex and % urban.

b Reference age category.

Sensitivity analyses conducted to assess the robustness of the findings (Supplementary Tables 1–3) did not change our conclusions. In NHS Direct there was no significant linear trend in IMD score; in NHS 111, GI calls significantly increased with increasing deprivation. In both NHS Direct and NHS 111, GI calls significantly increased with decreasing rurality. The results of analyses excluding calls regarding infants aged under 1 in NHS Direct and NHS 111 were comparable to the results including infants under 1 year of age. Including the mean distance to a GP as a variable in the main analysis did not alter our results (Supplementary Table 5).

## Discussion

In this nationally representative analysis of 24 million calls to NHS telephone helplines for health advice in England, there was a greater risk of GI calls from more disadvantaged areas compared to less disadvantaged areas. This is the only study of the relationship between SES and GI infection using telephone helpline data, which is the lowest level of healthcare interaction and the nearest to the population incidence for which we can get a full case ascertainment. The trend was most clear, increasing across all quintiles, in the NHS 111 dataset; in NHS Direct, there was a significantly lower risk of GI calls in the most disadvantaged children but no significant difference in risk for adults. There are a number of possible explanations for this difference. The introduction of NHS 111 in 2013 greatly increased the number of calls to the NHS helpline, including those for GI calls; however, whereas the proportion of non-GI calls from more disadvantaged areas decreased slightly, for GI calls the proportion from more deprived areas increased substantially. It could also relate to differences in the way individuals are interacting with NHS 111 compared to NHS Direct – this could be due to NHS 111 being a freephone number (which was not the case for NHS Direct) and representing a true gateway to unscheduled care as individuals will also use NHS 111 to access out of hours GP services whilst NHS Direct was a standalone telephone health system – although it is unclear why this would have a greater impact only upon calls for GI symptoms. It could reflect the lack of coded data for under 1 year olds from NHS Direct during the study period. In 2010/2011 there was a particularly high-level of influenza activity which may have affected the denominator for NHS Direct. Finally, one intriguing potential confounding factor is that the switch from NHS Direct to NHS111 coincided with the introduction of the rotavirus vaccine in young children, which led to a substantial reduction in the incidence of infectious intestinal disease^10^ and this could have had a differential effect by SES group, via uptake rates or effectiveness, as has been seen in other vaccine programmes.^11^^,^ ^12^ We were unable to disentangle these effects due to these events occurring almost contemporaneously.

### Strengths and limitations

A strength of this analysis is the novel use of two very large datasets of telephone advice calls to the NHS in England, not previously used to assess the social patterning of advice for GI illness. This dataset is the lowest level of healthcare interaction available, and therefore is as close to the true community incidence available from routinely collected data. Furthermore, entry into our study does not require an individual to present to formal healthcare settings nor have a sample taken and as such potentially represents a significant proportion of the GI infections which remain hidden from national surveillance systems. This is important if the decision to seek care is related to SES. A further strength was the ability to contrast the social patterning of GI calls with non-GI calls. Multiple sensitivity analyses were also undertaken to check the robustness of the findings and these corroborated our main conclusions. Despite being ecological, this study provides evidence of the existence of socioeconomic inequalities in GI infections. Community-level infection is important for GI infections due to person-to-person transmission and therefore it may actually be more appropriate to consider population-level analyses for this type of infection.

There are a number of limitations. Although this was a large study, and we were able to adjust for a number of potentially confounding variables, it is also possible that residual confounding, such as comorbidities, may remain. In addition, despite being nationally representative in terms of coverage, it is possible that the two datasets may not be representative of the population in terms of use of telephone helplines by SES. Furthermore, NHS Direct was under-representative of the elderly, who usually prefer to speak directly to a GP.^13^ Previous research conducted using data from NHS Direct has suggested that demand was highest in areas where deprivation was at or just above the national average,^14^ and that extreme deprivation appeared to raise adult call rates but reduce call rates in children.^14^ This could suggest a baseline difference in the demographics of the population interacting with this service but we were able to include postcode districts from which no calls originated as well as calculating crude incidence rates to compare callers in the context of the wider population at risk. This may also explain the findings for children in the NHS Direct dataset.

In addition, area-level measures of SES (each postcode district contains approximately 10,000 households), as used here, may not be sensitive enough to detect socioeconomic inequalities particularly where such inequalities are potentially generated by individual factors. As postcode district was the only available geographical measure for NHS Direct and NHS 111 calls, and as these may have crossed more than one geographical boundary, misclassification of SES is possible; however, we used population-weighted IMD scores to minimise this concern. A proportion of records for which there was no match to IMD score initially were manually cleaned whenever it was possible to identify the postcode district due to missing spaces, or letters substituted for numbers. This may have introduced the possibility of misclassification of IMD or the proportion of the population classed as urban. However, this affected only 0.1% (n = 14,639) of the total calls included in our analyses and is therefore unlikely to have affected the results. Similarly, postcode districts which bordered Scotland or Wales may have been misclassified, but this affected only a small number of postcode districts and is therefore unlikely to have caused a socioeconomic bias. The large size of this dataset is also likely to reduce these potential biases.

Syndromic surveillance systems monitor data that are not linked to specific pathogens or causes. In this study, GI symptoms were self-reported which may have resulted in some misclassification of the outcome (diarrhoea and/or vomiting). However, the routine use of clinical decision pathways by the telehealth service call-handlers to assess the presenting symptoms and determine the further healthcare needs of the patient is likely to have reduced the potential for misclassification and ensured consistency in the clinical decision making process between patients. It is also possible that the use of the clinical decision pathways resulted in the prioritisation of other presenting symptoms such as headache or fever over GI symptoms. In November 2011 NHS Direct changed the assessment protocol for infants aged less than 1 year which meant that symptom information was no longer available for syndromic surveillance. Nonetheless, our sensitivity analysis comparing results for NHS Direct including and excluding < 1 year olds demonstrated that this issue did not have an impact on the incidence rate ratios although it is not possible to know what the impact on the results would be if this data were available for the whole period. Further, the analyses forming this study are cross-sectional and as such, it is not possible to determine causation.

### Comparison with previous studies

One study by Cooper et al.^15^ exploring 150,000 GI calls to NHS Direct over a six-month period at three sites found that GI calls accounted for 10.3% of total calls; this proportion was significantly higher among children under 1 year of age (23.5%) and aged 1-4 years (21.5%). This finding is slightly higher than the 6% of calls in our study being classed as GI calls. This study did not explore socioeconomic inequalities in GI calls.

Several studies have explored the social patterning of calls to NHS Direct, but not specifically for GI calls. Cooper et al. ^14^ used NHS Direct calls to assess socio-demographic patterning. As found in the study mentioned above, calls were highest in children under five years of age and were higher in women compared to men; with the highest ratio in the 15–44 year age group. The authors found that the effect of extreme deprivation appeared to raise adult call rates but reduce call rates for children. This is similar to the findings in our study for non-GI calls. The study by Cooper et al. was conducted on all calls, without distinction between GI and non-GI calls, and covered only two regions of England; the authors recommend further national studies are undertaken to validate their findings. In our study, higher rates of GI calls in more deprived compared to less disadvantaged areas were observed overall and for children in NHS 111, which is a novel finding, and complements the results of the study by Cooper et al.

Burt et al. ^16^ found that there was a significant non-linear relationship between deprivation score and call rates to NHS Direct, with lower rates in the most affluent and the most disadvantaged areas of London. The authors suggest that the decline at the extremes of deprivation scores may reflect barriers to accessing NHS Direct. There is a very high ethnic minority proportion in London, particularly in disadvantaged areas, and this may have impacted on the results if language is a barrier to using telephone-based services. In our study, we found that rates peaked in quintile 4 in NHS Direct and reduced slightly in quintile 5 (most disadvantaged).

Shah and Cook ^17^ found that NHS Direct use was lower in households with low income (OR 0.67; 95% CI 0.55-0.81); adjusting for limiting illness increased the effect of socioeconomic factors on NHS Direct use. Qualitative studies have also been used to explore the social patterning of callers to NHS Direct. Cook et al. ^13^ used focus groups with users and non-users of NHS Direct to explore barriers to use. The authors found that there were a range of barriers including the cost of making a phone call to NHS Direct and that this view was expressed more often by non-users from disadvantaged communities. The NHS 111 system is free to call although the authors highlighted that this change should be clearly communicated to the general public to increase awareness and use. In our study, we found significantly higher risk of GI calls amongst the most disadvantaged compared to the least disadvantaged for both NHS Direct and NHS 111, although the risk ratios were lower in the NHS Direct dataset. In addition, call volume greatly increased following the introduction of NHS 111; this was particularly evident for GI calls in the most disadvantaged areas which may reflect NHS 111 being used also as an out-of-hours services.

There are several possible explanations for the finding of higher odds of calls regarding GI symptoms amongst more disadvantaged individuals in this study. The finding may be artefactual; the study population may not be representative of the general population and may differ from the population not using NHS telephone-based healthcare advice services. However, the sample was large, the findings consistent across groups, and the internal associations, which were the targets of inference within the sample population, are likely to be valid. Moreover the inclusion of postcode districts from which no calls were received enabled us to take account of the underlying population at risk. On the other hand, it could also be that more disadvantaged individuals have a genuinely higher risk of GI symptoms compared to less disadvantaged individuals. This may relate to differential exposure, differential vulnerability to disease, or reflect differences in the recognition or reporting of symptoms or differential healthcare seeking behaviour by SES.

In summary, this study provides new evidence of a relationship between GI infections and SES. Amongst people calling NHS telephone-based healthcare advice services, people from more disadvantaged areas were more likely to call NHS 111 for GI symptoms compared to people calling from less disadvantaged areas, and this relationship is stronger than that for non-GI calls and held for all age-groups. In NHS Direct, there was a significantly lower risk of a GI call for children from the most disadvantaged areas, compared to the least, but no difference for adults which could relate to important differences between the two systems. This finding has implications for service providers and the NHS in terms of resource allocation in disadvantaged areas where call volume to NHS 111 may be higher particularly for GI infections. Further research is required to explore the role of symptom recognition, perception, healthcare interaction and other potentially mediating exposures to complement these results and help to explain the relationship between SES and GI infection in more depth. A greater understanding of the individual behaviours and risk factors by SES is crucial to understanding the differential risk, vulnerability, and consequences of GI infections. Our results contribute to the evidence on community-level risk of and vulnerability to GI infections amongst individuals seeking care through NHS telephone-based healthcare advice services. Alongside future planned analyses, these results could ultimately be used to provide further evidence to inform policies to address inequalities in risk, vulnerability and consequences of GI infections.

## Funding

The research was funded by the National Institute for Health Research Health Protection Research Unit (NIHR HPRU) in Gastrointestinal Infections at University of Liverpool in partnership with Public Health England (PHE), in collaboration with University of East Anglia, University of Oxford and the Quadram Institute. Natalie Adams is based at the University of Liverpool and Public Health England. The views expressed are those of the author(s) and not necessarily those of the NHS, the NIHR, the Department of Health and Social Care or Public Health England. David Taylor-Robinson is funded by the MRC on a Clinician Scientist Fellowship (MR/P008577/1). Ben Barr and Tanith Rose were also supported by the National Institute for Health Research (NIHR) Collaboration for Leadership in Health Research and Care (CLAHRC NWC).

## Disclaimers

The views expressed are those of the author(s) and not necessarily those of the NHS, the NIHR, the Department of Health and Social Care or Public Health England.

## Conflict of interest

The authors declare that they have no conflicts of interest.

## Appendix Supplementary materials

Image, application 1
