# Supplementary material for: Social patterning of telephone health-advice for diarrhoea and vomiting: analysis of 24 million telehealth calls in England
Source: J Infect. 2019 Feb;78(2):95–100. doi: 10.1016/j.jinf.2018.09.008 (PMC6428660; doi:10.1016/j.jinf.2018.09.008)
Supplement: Supplementary file 1 [file mmc1.docx]

**SUPPLEMENTARY DATA**

Supplementary Table 1: Univariate and multivariable regression analysis GI calls – Sensitivity postcode districts with population=0 recoded to population=1 by system

| **Variable** | **Category** | **Univariate** | **Multivariable^a^** | **p value** |
| --- | --- | --- | --- | --- |
|  |  | **RR (95% CI)** | **RR (95% CI)** |  |
| **NHS Direct (n=25,008)** | | | | |
| **IMD Quintile** | 1 (least disadvantaged) | 1.00 (reference) | 1.00 (reference) |  |
|  | 2 | 1.00 (0.99-1.01) | 1.00 (0.99-1.01) | 1.00 |
|  | 3 | 1.04 (1.03-1.05) | 0.99 (0.98-1.01) | 1.00 |
|  | 4 | 1.10 (1.09-1.11) | 0.97 (0.96-0.98) | <0.001 |
|  | 5 (most disadvantaged) | 1.07 (1.05-1.08) | 0.88 (0.87-0.89) | <0.001 |
| **Age Group** | 0-4 | 8.17 (8.12-8.22) | 8.19 (8.14-8.24) | <0.001 |
|  | 5-9 | 1.06 (1.06-1.07) | 1.06 (1.05-1.08) | <0.001 |
|  | 10-14 | 0.51 (0.50-0.52) | 0.51 (0.50-0.52) | <0.001 |
|  | 15-19 | 1.12 (1.11-1.14) | 1.13 (1.11-1.14) | <0.001 |
|  | 20-59 | 1.00 (reference) | 1.00 (reference) |  |
|  | 60+ | 0.95 (0.94-0.96) | 0.95 (0.94-0.96) | <0.001 |
| **Sex** | Male | 1.00 (reference) | 1.00 (reference) |  |
|  | Female | 1.28 (1.27-1.28) | 1.31 (1.30-1.32) | <0.001 |
| **Urban decile (%)** | <10 | 0.72 (0.71-0.72) | 0.75 (0.75-0.76) | <0.001 |
|  | 10-19 | 0.77 (0.76-0.79) | 0.80 (0.78-0.81) | <0.001 |
|  | 20-29 | 0.82 (0.81-0.84) | 0.85 (0.83-0.86) | <0.001 |
|  | 30-39 | 0.83 (0.82-0.85) | 0.85 (0.83-0.86) | <0.001 |
|  | 40-49 | 0.87 (0.85-0.88) | 0.90 (0.88-0.91) | <0.001 |
|  | 50-59 | 0.86 (0.84-0.87) | 0.87 (0.85-0.88) | <0.001 |
|  | 60-69 | 0.91 (0.90-0.93) | 0.92 (0.91-0.93) | <0.001 |
|  | 70-79 | 0.97 (0.95-0.98) | 0.98 (0.97-1.00) | 0.05 |
|  | 80-89 | 0.95 (0.93-0.96) | 0.95 (0.94-0.96) | <0.001 |
|  | 90-100 | 1.00 (reference) | 1.00 (reference) |  |
| **NHS 111 (n=25,008)** | | | | |
| **IMD Quintile** | 1 (least disadvantaged) | 1.00 (reference) | 1.00 (reference) |  |
|  | 2 | 1.19 (1.18-1.20) | 1.19 (1.18-1.20) | <0.001 |
|  | 3 | 1.39 (1.38-1.41) | 1.36 (1.35-1.37) | <0.001 |
|  | 4 | 1.46 (1.45-1.47) | 1.35 (1.34-1.36) | <0.001 |
|  | 5 (most disadvantaged) | 1.50 (1.49-1.52) | 1.32 (1.31-1.33) | <0.001 |
| **Age Group** | 0-4 | 11.31 (11.26-11.36) | 11.32 (11.34-11.38) | <0.001 |
|  | 5-9 | 1.24 (1.23-1.25) | 1.25 (1.24-1.26) | <0.001 |
|  | 10-14 | 0.54 (0.54-0.55) | 0.55 (0.54-0.59) | <0.001 |
|  | 15-19 | 1.34 (1.33-1.35) | 1.35 (1.33-1.36) | <0.001 |
|  | 20-59 | 1.00 (reference) | 1.00 (reference) |  |
|  | 60+ | 1.58 (1.57-1.59) | 1.58 (1.58-1.59) | <0.001 |
| **Sex** | Male | 1.00 (reference) | 1.00 (reference) |  |
|  | Female | 1.37 (1.36-1.38) | 1.46 (1.45-1.46) | <0.001 |
| **Urban decile (%)** | <10 | 0.73 (0.73-0.74) | 0.84 (0.83-0.85) | <0.001 |
|  | 10-19 | 0.84 (0.83-0.85) | 0.96 (0.94-0.97) | <0.001 |
|  | 20-29 | 0.84 (0.83-0.85) | 0.92 (0.91-0.94) | <0.001 |
|  | 30-39 | 0.91 (0.90-0.92) | 0.97 (0.96-0.99) | <0.001 |
|  | 40-49 | 0.93 (0.92-0.94) | 1.02 (1.01-1.04) | <0.001 |
|  | 50-59 | 0.88 (0.87-0.90) | 0.94 (0.93-0.95) | <0.001 |
|  | 60-69 | 0.83 (0.82-0.84) | 0.89 (0.88-0.90) | <0.001 |
|  | 70-79 | 0.95 (0.94-0.95) | 0.97 (0.96-0.98) | <0.001 |
|  | 80-89 | 0.94 (0.94-0.95) | 0.99 (0.98-1.00) | 0.05 |
|  | 90-100 | 1.00 (reference) | 1.00 (reference) |  |
| RR – Risk Ratio; ^a^ Adjusted for all other covariates in the model | | | | |

Supplementary Table 2: Univariate and multivariable regression analysis GI-calls – Sensitivity IMD score and proportion of population classed as urban as continuous variables by system

| **Variable** | **Category** | **Univariate** | **Multivariable^a^** | **p value** |
| --- | --- | --- | --- | --- |
|  |  | **RR (95% CI)** | **RR (95% CI)** |  |
| **NHS Direct (n=24,985)** | | | | |
| **IMD Score** |  | 1.00 (1.00-1.00) | 1.00 (1.00-1.00) | <0.001 |
| **Age Group** | 0-4 | 8.17 (9.12-8.22) | 8.19 (8.14-8.24) | <0.001 |
|  | 5-9 | 1.06 (1.04-1.07) | 1.06 (1.05-1.08) | <0.001 |
|  | 10-14 | 0.51 (0.50-0.52) | 0.51 (0.50-0.52) | <0.001 |
|  | 15-19 | 1.12 (1.11-1.14) | 1.13 (1.11-1.14) | <0.001 |
|  | 20-59 | 1.00 (reference) | 1.00 (reference) |  |
|  | 60+ | 0.95 (0.94-0.96) | 0.95 (0.94-0.96) | <0.001 |
| **Sex** | Male | 1.00 (reference) | 1.00 (reference) |  |
|  | Female | 1.28 (1.27-1.28) | 1.31 (1.30-1.32) | <0.001 |
| **Urban (%)** |  | 1.00 (1.00-1.00) | 1.00 (1.00-1.00) | <0.001 |
| **NHS 111 (n=24,985)** | | | | |
| **IMD Score** |  | 1.01 (1.01-1.01) | 1.00 (1.00-1.00) | <0.001 |
| **Age Group** | 0-4 | 11.31 (11.26 -11.36) | 11.30 (11.25-11.36) | <0.001 |
|  | 5-9 | 1.24 (1.23-1.25) | 1.25 (1.23-1.26) | <0.001 |
|  | 10-14 | 0.54 (0.54-0.55) | 0.55 (0.54-0.57) | <0.001 |
|  | 15-19 | 1.34 (1.33-1.35) | 1.35 (1.33-1.36) | <0.001 |
|  | 20-59 | 1.00 (reference) | 1.00 (reference) |  |
|  | 60+ | 1.58 (1.57-1.59) | 1.59 (1.58-1.60) | <0.001 |
| **Sex** | Male | 1.00 (reference) | 1.00 (reference) |  |
|  | Female | 1.37 (1.36-1.38) | 1.40 (1.39-1.40) | <0.001 |
| **Urban (%)** |  | 1.00 (1.00-1.00) | 1.00 (1.00-1.00) | <0.001 |
| RR – Risk Ratio; ^a^ Adjusted for all other covariates in the model | | | | |

Supplementary Table 3a: Univariate and multivariable regression analysis GI calls – Sensitivity analysis <1 year olds excluded NHS Direct (n=24,985)

| **Age group** | **IMD Quintile** | **RR^a^ (95% CI)** | **p value** |
| --- | --- | --- | --- |
| **0-4** | **1** (Least disadvantaged) | 1.00 (reference) |  |
|  | **2** | 1.02 (1.00-1.04) | <0.001 |
|  | **3** | 1.01 (0.99-1.03) | <0.001 |
|  | **4** | 0.97 (0.95-0.98) | <0.001 |
|  | **5** (Most disadvantaged) | 0.83 (0.82-0.85) | <0.001 |
| ^a^Adjusted for sex, % urban | | | |

Supplementary Table 3b: Rates per 10,000 person-months and incidence rate ratio for exposed (most disadvantaged) compared to unexposed (least disadvantaged) in NHS Direct by age group Sensitivity analysis <1 year olds included compared to <1 year olds excluded

|  | **<1s Included** | **<1s Excluded** |
| --- | --- | --- |
| **GI calls** | | |
| Overall rate 0-4 | 16.5 | 11.5 |
| 0-4 Rate/10,000* in most disadvantaged | 14.1 | 9.8 |
| 0-4 Rate/10,000* in least disadvantaged | 17.7 | 12.2 |
| Incidence rate ratio (95% CI) | 0.79 (0.78-0.81) | 0.80 (0.78-0.82) |
| Overall rate 5+ | 2.0 | 2.0 |
| 5+ Rate/10,000* in most disadvantaged | 2.0 | 2.0 |
| 5+ Rate/10,000* in least disadvantaged | 1.8 | 1.8 |
| Incidence rate ratio (95% CI) | 1.10 (1.08-1.12) | 1.10 (1.08-1.12) |
| **Non-GI calls** | | |
| Overall rate 0-4 | 132.7 | 86.0 |
| 0-4 Rate/10,000* in most disadvantaged | 112.4 | 71.2 |
| 0-4 Rate/10,000* in least disadvantaged | 146.3 | 96.4 |
| Incidence rate ratio (95% CI) | 0.77 (0.76-0.77) | 0.74 (0.73-0.75) |
| Overall rate 5+ | 33.3 | 33.3 |
| 5+ Rate/10,000* in most disadvantaged | 37.6 | 37.6 |
| 5+ Rate/10,000* in least disadvantaged | 29.3 | 29.3 |
| Incidence rate ratio (95% CI) | 1.28 (1.28-1.29) | 1.28 (1.28-1.29) |
| *Person-months; GI – Gastrointestinal infection; CI – Confidence interval; NHS – National Health Service | | |

Supplementary Table 4: Crude rates of calls per 10,000 person-months by system

|  | **1 (Least Disadvantaged)** | **2** | **3** | **4** | **5 (Most Disadvantaged)** |
| --- | --- | --- | --- | --- | --- |
| **NHS Direct** | | | | | |
| **GI-calls overall** | 2.7 | 2.7 | 2.8 | 3.0 | 2.9 |
| Female | 3.0 | 3.0 | 3.2 | 3.4 | 3.3 |
| Male | 2.4 | 2.4 | 2.5 | 2.6 | 2.5 |
| 0-4 | 17.7 | 17.5 | 17.3 | 16.5 | 14.1 |
| 5-9 | 2.1 | 2.1 | 2.2 | 2.2 | 2.0 |
| 10-14 | 0.9 | 1.0 | 1.1 | 1.1 | 0.9 |
| 15-19 | 2.0 | 2.1 | 2.3 | 2.4 | 2.3 |
| 20-59 | 1.9 | 1.9 | 2.0 | 2.1 | 2.1 |
| 60+ | 1.8 | 1.8 | 1.9 | 2.0 | 2.0 |
| **Non GI-calls overall** | 36.1 | 36.6 | 38.5 | 41.4 | 43.3 |
| Female | 41.3 | 41.9 | 44.5 | 48.2 | 50.7 |
| Male | 30.7 | 31.0 | 32.4 | 34.3 | 35.7 |
| 0-4 | 146.3 | 142.7 | 138.7 | 129.7 | 112.4 |
| 5-9 | 26.7 | 26.5 | 26.9 | 26.1 | 23.7 |
| 10-14 | 17.5 | 17.5 | 18.2 | 17.9 | 16.7 |
| 15-19 | 34.1 | 36.0 | 39.4 | 43.3 | 46.0 |
| 20-59 | 32.4 | 33.5 | 35.9 | 39.3 | 42.7 |
| 60+ | 25.7 | 26.1 | 27.3 | 28.8 | 29.7 |
| **NHS 111** | | | | | |
| **GI-calls overall** | 5.4 | 6.4 | 7.5 | 7.8 | 8.1 |
| Female | 6.2 | 7.4 | 8.6 | 9.0 | 9.3 |
| Male | 4.5 | 5.3 | 6.3 | 6.6 | 6.9 |
| 0-4 | 34.9 | 42.1 | 49.0 | 48.7 | 46.7 |
| 5-9 | 3.7 | 4.3 | 5.2 | 5.6 | 5.6 |
| 10-14 | 1.7 | 2.0 | 2.3 | 2.4 | 2.4 |
| 15-19 | 3.7 | 4.6 | 5.6 | 6.0 | 6.3 |
| 20-59 | 2.9 | 3.6 | 4.2 | 4.4 | 4.5 |
| 60+ | 5.4 | 6.1 | 6.9 | 6.7 | 6.4 |
| **Non GI-calls overall** | 118.3 | 121.6 | 129.6 | 134.1 | 125.0 |
| Female | 133.6 | 137.8 | 147.0 | 153.1 | 143.0 |
| Male | 102.4 | 104.8 | 111.4 | 114.5 | 106.7 |
| 0-4 | 381.8 | 384.5 | 383.7 | 355.2 | 289.9 |
| 5-9 | 91.5 | 91.8 | 94.8 | 90.9 | 76.0 |
| 10-14 | 55.0 | 55.2 | 55.8 | 55.4 | 49.1 |
| 15-19 | 104.5 | 110.9 | 123.7 | 133.6 | 131.2 |
| 20-59 | 91.5 | 95.9 | 105.4 | 113.6 | 113.3 |
| 60+ | 137.7 | 139.8 | 147.2 | 152.4 | 133.9 |

Supplementary Table 5: Multivariable regression analysis presenting main effect with interaction terms for GI calls in each age group by system, adjusted for average distance to GP

|  |  | **NHS Direct** | **NHS 111** |
| --- | --- | --- | --- |
| **Age group** | **IMD Quintile** | **RR^a^ (95% CI)** | **RR^a^ (95% CI)** |
| **0-4** | **1** (Least disadvantaged) | 1.00 (reference) | 1.00 (reference) |
|  | **2** | **0.98 (0.96-1.00)** | **1.20 (1.18-1.22)** |
|  | **3** | **0.93 (0.92-0.95)** | **1.37 (1.35-1.39)** |
|  | **4** | **0.84 (0.83-0.86)** | **1.33 (1.31-1.35)** |
|  | **5** (Most disadvantaged) | **0.70 (0.69-0.72)** | **1.26 (1.24-1.28)** |
| **5-9** | **1** (Least disadvantaged) | 1.00 (reference) | 1.00 (reference) |
|  | **2** | 0.98 (0.93-1.04) | **1.16 (1.11-1.22)** |
|  | **3** | 1.01 (0.96-1.07) | **1.38 (1.32-1.45)** |
|  | **4** | 0.95 (0.90-1.00) | **1.43 (1.37-1.50)** |
|  | **5** (Most disadvantaged) | **0.85 (0.80-0.90)** | **1.42 (1.35-1.49)** |
| **10-14** | **1** (Least disadvantaged) | 1.00 (reference) | 1.00 (reference) |
|  | **2** | **1.09 (1.00-1.18)** | **1.19 (1.11-1.28)** |
|  | **3** | **1.11 (1.03-1.21)** | **1.36 (1.27-1.45)** |
|  | **4** | 1.06 (0.98-1.15) | **1.36 (1.27-1.45)** |
|  | **5** (Most disadvantaged) | **0.90 (0.82-0.98)** | **1.34 (1.25-1.45)** |
| **15-19** | **1** (Least disadvantaged) | 1.00 (reference) | 1.00 (reference) |
|  | **2** | **1.06 (1.01-1.13)** | **1.24 (1.18-1.30)** |
|  | **3** | **1.12 (1.06-1.18)** | **1.48 (1.41-1.55)** |
|  | **4** | **1.10 (1.04-1.16)** | **1.54 (1.47-1.61)** |
|  | **5** (Most disadvantaged) | 1.02 (0.96-1.08) | **1.58 (1.50-1.65)** |
| **20-59 ^b^** | **1** (Least disadvantaged) | 1.00 (reference) | 1.00 (reference) |
|  | **2** | **1.09 (1.00-1.18)** | **1.23 (1.21-1.26)** |
|  | **3** | **1.11 (1.03-1.21)** | **1.42 (1.40-1.45)** |
|  | **4** | 1.06 (0.98-1.15) | **1.46 (1.43-1.48)** |
|  | **5** (Most disadvantaged) | **0.90 (0.82-0.98)** | **1.49 (1.46-1.51)** |
| **60+** | **1** (Least disadvantaged) | 1.00 (reference) | 1.00 (reference) |
|  | **2** | 1.00 (0.97-1.03) | **1.13 (1.11-1.15)** |
|  | **3** | **1.03 (1.00-1.06)** | **1.26 (1.24-1.28)** |
|  | **4** | 1.02 (0.99-1.05) | **1.19 (1.17-1.21)** |
|  | **5** (Most disadvantaged) | 0.97 (0.94-1.00) | **1.11 (1.08-1.13)** |
| ^a^Linear combination of main effect + interaction between age and IMD quintile, adjusted for sex, % urban and average distance to GP; ^b^Reference age category  GI – Gastrointestinal infection; CI – Confidence interval; NHS – National Health Service; GP – General Practice | | | |
